# Ectopic Expression of Ankrd2 Affects Proliferation, Motility and Clonogenic Potential of Human Osteosarcoma Cells

**DOI:** 10.3390/cancers13020174

**Published:** 2021-01-06

**Authors:** Manuela Piazzi, Snezana Kojic, Cristina Capanni, Nemanja Stamenkovic, Alberto Bavelloni, Oriano Marin, Giovanna Lattanzi, William Blalock, Vittoria Cenni

**Affiliations:** 1 Unit of Bologna, Institute of Molecular Genetics “Luigi Luca Cavalli-Sforza”, National Research Council of Italy (IGM-CNR), 40136 Bologna, Italy; manuela.piazzi@cnr.it (M.P.); ccapanni@area.bo.cnr.it (C.C.); giovanna.lattanzi@cnr.it (G.L.); 2 IRCCS, Istituto Ortopedico Rizzoli, 40136 Bologna, Italy; 3 Institute of Molecular Genetics and Genetic Engineering, University of Belgrade, 11042 Belgrade, Serbia; snezanakojic@imgge.bg.ac.rs (S.K.); stamenkovicnemanja22@gmail.com (N.S.); 4 Laboratory of Experimental Oncology, IRCCS Istituto Ortopedico Rizzoli, 40136 Bologna, Italy; alberto.bavelloni@ior.it; 5 Department of Biomedical Sciences, University of Padua, 35131 Padua, Italy; oriano.marin@unipd.it

**Keywords:** Ankrd2, cancer, osteosarcoma, bone, proliferation, mechanotransduction, migration, spheroids

## Abstract

**Simple Summary:**

Osteosarcoma is a rare malignancy of bone, primarily affecting children and young adults. The main objective of this study was to identify novel therapeutic targets to fight the progression of this insidious disease. To this aim, the role of Ankrd2, a stress- and mechano- sensor protein known for being mostly expressed in muscle fibers, was analyzed in the modulation of osteosarcoma progression. By subjecting human osteosarcoma cell lines expressing or silencing Ankrd2 to several functional assays, our results demonstrated that Ankrd2 is involved in the pathogenesis of this cancer. Nonetheless, due to observations obtained by other studies in other model systems, our findings also suggest that Ankrd2 might behave as a “double-faced” cancer driver gene.

**Abstract:**

Ankrd2 is a protein known for being mainly expressed in muscle fibers, where it participates in the mechanical stress response. Since both myocytes and osteoblasts are mesenchymal-derived cells, we were interested in examining the role of Ankrd2 in the progression of osteosarcoma which features a mechano-stress component. Although having been identified in many tumor-derived cell lines and -tissues, no study has yet described nor hypothesized any involvement for this protein in osteosarcoma tumorigenesis. In this paper, we report that Ankrd2 is expressed in cell lines obtained from human osteosarcoma and demonstrate a contribution by this protein in the pathogenesis of this insidious disease. Ankrd2 involvement in osteosarcoma development was evaluated in clones of Saos2, U2OS, HOS and MG63 cells stably expressing Ankrd2, through the investigation of hallmark processes of cancer cells. Interestingly, we found that exogenous expression of Ankrd2 influenced cellular growth, migration and clonogenicity in a cell line-dependent manner, whereas it was able to improve the formation of 3D spheroids in three out of four cellular models and enhanced matrix metalloproteinase (MMP) activity in all tested cell lines. Conversely, downregulation of Ankrd2 expression remarkably reduced proliferation and clonogenic potential of parental cells. As a whole, our data present Ankrd2 as a novel player in osteosarcoma development, opening up new therapeutic perspectives.

## 1. Introduction

Ankrd2 (Ankyrin repeat domain 2), also known as Arpp (Ankyrin Repeat protein with PEST and Proline-rich region), belongs to the MARP (Muscle Ankyrin Repeat Protein) family, which also includes Ankrd1/CARP and Ankrd23/DARP [1,2]. MARP proteins share several functional domains, including ankyrin repeats, involved in protein-protein interaction, PEST motifs, which are regions of protein instability, and a nuclear localization signal (NLS) for nuclear translocation of the protein [1]. The MARPs are mainly found in cardiac and skeletal muscle, although Ankrd1 is mostly expressed in the heart [3] and Ankrd2 in skeletal muscle [2,3,4]. There are two Ankrd2 isoforms detected in striated muscle, namely S- and M- Ankrd2 [5]. They are identical, except for a 27 amino acid extension at the N-terminus of M-Ankrd2 [5,6]. The expression of Ankrd2 isoforms was found to be regulated at the transcriptional level [5]. Being the most represented one, S-Ankrd2 has been proposed as the canonical one; this also happens to be the isoform our group found to be most expressed in osteosarcoma cells, which we report here.

In muscle cells, Ankrd2 is considered an intracellular downstream effector of signal transduction pathways elicited in response to mechanical and stress stimuli, thus acting as a mechanotransducer or a mechanosensor depending on the biological context [6,7,8]. In fact, multiple lines of evidence support a role for Ankrd2 as an important regulator of myogenesis. During the earlier phases of myogenesis, Ankrd2 expression, which is under the control of MyoD, facilitates the exit of myoblasts from the cell cycle via inducing the p53-dependent pathway [9]. However, after the early phases of myogenesis, Ankrd2 accumulation impairs muscle differentiation by downregulating the transcription of MyoD and myogenin [9]. Findings from our laboratory suggest that Ankrd2 plays an active role in the modulation of myogenesis upon oxidative stress conditions [10]. We demonstrated that upon the exposure to oxidative stress, Ankrd2 is phosphorylated by Akt2 on serine 99 (S99), resulting in negative effects on muscle differentiation [10]. Our studies, in fact, revealed that the overexpression of an unphosphorylatable form of Ankrd2 decreased the differentiation rate of Ankrd2 wild-type transfected cells [10]. Studies performed in primary proliferating or differentiating myoblasts have further revealed that the ectopic expression of Ankrd2 acts as a potent repressor of the inflammatory response [11]. Additionally, in this case, the lack of phosphorylation on S99 was able to preclude the anti-inflammatory effects of Ankrd2 [11].

Osteosarcoma (OS) is the most common type of primary malignant bone tumor, mainly occurring in childhood and in the second decade of life during the adolescent growth spurt [12]. Non metastatic-OS can be treated with surgery, radiotherapy and chemotherapy, while pulmonary metastases remain the most important fatal complication of OS [13]. Pharmacological treatment of OS requires chemotherapeutic drugs, such as doxorubicin, cisplatin, cyclophosphamide, methotrexate and etoposide, often used in combination [14,15]. Due to the high risk of metastasis, recurrence and chemoresistance, there is a clear need to discover novel therapeutic targets and effective agents for treating patients affected by this disease. As published evidence has reported that Ankrd2 is also expressed in tumor cells, such as human esophageal carcinoma [2] and rhabdomyosarcoma [3,16], and since osteoblasts and myocytes are mesenchymal-derived cells strongly susceptible to mechanical stress, in order to discover new therapeutic targets, the main object of this study was to evaluate the role of Ankrd2 in the patho-physiology of OS. Our results demonstrated that the manipulation of the Ankrd2 expression level affected cellular proliferation, migration, clonogenic potential and anchorage-independent growth of OS cell lines, hinting at a role for Ankrd2 in tumor progression not previously described.

## 2. Materials and Methods

### 2.1. Cultures, Transfection and Cellular Treatments

Cell lines from human osteosarcoma (Saos2, U2OS, MG63 and HOS), from normal human osteoblasts (hFOB 1.19) and from human rhabdomyosarcoma (RMS) (RD, HS729, SJCRH30 and A204) were obtained from ATCC-LGC Standards Srl, Milan, Italy. OS cells were cultured in Iscove’s Modified Dulbecco’s Medium (IMDM) GlutaMAX (Gibco, ThermoFisher Scientific, Monza, Italy) supplemented with 10% of heat-inactivated fetal bovine serum (FBS, Gibco). RMS cells were cultured in Dulbecco’s Modified Eagle Medium (DMEM) Glutamax (Gibco) supplemented with 10% FBS. Cells were maintained in a humidified atmosphere with 5% CO_2_ at 37 °C and subcultured twice a week. hFOB cells were cultured in DMEM/Nutrient Mixture F-12 Ham (Gibco) supplemented with 10% FBS and L-glutamine. Cells were maintained in a humidified atmosphere with 5% CO_2_ at 34 °C and subcultured once a week. Stable clones of hFOB, RD, U2OS, MG63, HOS and Saos2 expressing Flag-Ankrd2wt, Flag-Ankrd2(S99A) or vector control (EV) were obtained by transfecting cells with the pcDNA3 eukaryotic expression vector encoding Flag-tagged Ankrd2wt, Flag-tagged Ankrd2(S99A) or vector alone, respectively, with Fugene6 (Promega, Madison, WI, USA) and selection in the presence of G418 (Sigma-Aldrich, St. Louis, MO, USA), at a concentration of 600 μg/mL for U2OS and HOS, and 400 μg/mL for hFOB, MG63, RD and Saos2 cells. For U2OS and HOS cells, Ankrd2-silenced clones were obtained by transfecting cells with the pSUPER.neo+GFP (OligoEngine, Seattle, WA, USA) eukaryotic expression vector and selected with G418 as described below. After one month of selection, clones were isolated by trypsinization and separately cultured.

### 2.2. Plasmids

Flag-tagged human Ankrd2wt (S-isoform) and Ankrd2(S99A) have been previously described [10] and were inserted into the pcDNA3 eukaryotic expression vector. Cloning primers were: 5′-CGGGGTACCGCCATGGACTACAAGGACGAC and 3′-ATATTAGGGCCCTCACTGGGCTGGCACAGG (KpnI-ApaI cloning sites). Cloning and mutagenic primers were purchased from IDT technologies (Tema Ricerca Srl, Castenaso, Italy). Plasmids were verified by a bi-directional DNA sequencing service (BMR, Padua, Italy).

### 2.3. Stable Silencing

For stable Ankrd2 silencing, U2OS and HOS cells were transfected with pSUPER.neo+GFP expression vectors encoding siRNA-like transcripts targeting exon 1 and 2 of the human Ankrd2 gene. Oligos for creating si-Ankrd2 transcripts were: 5′-GATCCCCGGAGGAGGAGAATGAGAAATTCAAGAGATTTCTCATTCTCCTCCTCC TTTTTA-3′; 3′-GGGCCTCCTCCTCTTACTCTTTAAGTTCTCTAAAGAGTAAGAGGAG GAGGAAAAATTCGA-5′, in which underlined sequences are those targeting Ankrd2 transcript. Control non-targeting siRNA sequences were obtained by inserting the following oligos: 5′-GATCCCCGACGAAAGCAGTTCGTCTATTCAAGAGATAGACGAACTGCTTT CGTCTTTTTA-3′ and 5′-AGCTTAAAAAGACGAAAGCAGTTCGTCTATCTCTTGAA TAGACGAACTGCTTTCGTCGGG-3′, in which underlined sequences correspond to a scramble RNA sequence validated against the human genome.

### 2.4. RT-PCR and qPCR

Total RNA was isolated from 60%-confluence cultured cells using TRIzol reagent (Thermo Fisher Scientific, Waltham, MA, USA) according to the manufacturer’s protocol. RNA concentration and purity were measured by a Ultrospec 3300 Pro spectrophotometer (Amersham Biosciences, Little Chalfont, UK). Random hexamer-primed cDNA was generated by reverse transcription, using 2 μg of total RNA and the High Capacity cDNA Reverse Transcription Kit (Thermo Fisher Scientific, Waltham, MA, USA), according to the manufacturer’s instructions. *ANKRD2* and *GAPDH* fragments were amplified using Native Taq DNA polymerase (EURx, Gdansk, Poland), cDNA as a template and primers listed in Table S1. The PCR reaction was performed in a 2720 Thermal Cycler (Applied Biosystems, Foster City, CA, USA), under the following conditions: 1 cycle of 95 °C for 3 min; 35 cycles for *ANKRD2* or 30 cycles for *GAPDH* of 95 °C for 30 s, 60 °C for 35 s and 72 °C for 30 s; followed by 1 cycle of 72 °C for 10 min. *GAPDH* was used as cDNA quality control.

Quantitative PCR (qPCR) was performed on a 7900HT Fast Real-Time System (Applied Biosystems, Foster City, CA, USA), in technical triplicate for each sample, and for at least three biological replicates for each cell line. The reaction profile for amplification of *ANKRD2* and *GAPDH* fragments, using primers listed in Table S1, and Power SYBR Green PCR Master Mix (Applied Biosystems, Foster City, CA, USA), was as follows: initial denaturation at 95 °C for 10 min, 40 cycles of denaturation at 95 °C for 15 s and annealing and elongation at 60 °C for 60 s. Amplification was followed by the melting curve/dissociation analysis. The *GAPDH* transcript served as an internal reference to normalize the mRNA levels in different samples. The qPCR data were analyzed using the 2^−ΔCt^ method. Statistical analysis was performed by paired two-tail Student’s *t*-test. Results were presented as mean ± SD; the level of significance was *p* < 0.01.

### 2.5. Cell Proliferation/Viability

From 2 to 3 × 10^5^ cells were seeded in each well of six-well tissue culture plates. Cells in the respective well were trypsinized and collected at 1, 2 and 3 days after plating. Cells were stained with trypan blue (Sigma), and living and dead cells counted with a Neubauer haemocytometry. The percent viability (%V) was calculated with the canonical formula: %V = (live cells/total cells) × 100. Results were graphed and analyzed by Prism v5.0 (GraphPad software, San Diego, CA, USA).

### 2.6. Cell Motility Assays

- Wound healing assay: Cells were seeded at 70% of confluence. Once confluence was reached, three wounds (“scratches”) were applied to the monolayer using a 200 µL pipette tip. The wounds were observed and captured in triplicate at the time of wounding (T0) and after 7, 24 and 48 h, under light microscopy using a microscope (Carl Zeiss, Munich, Germany) equipped with an AxioVision digital camera. The width of the scratches was measured using the AxioVision Rel 4.7 Software and the percentage (%) of the wound closure calculated according the formula: *((Tn-T0)/T0)* × 100, where *T0* is the width of the scratch at the beginning and *Tn* is the width at 7, 24 and 48 h after the scratch.
- Transwell migration assay: Sub-confluent flasks of Ankrd2-expressing OS-derived cells and their respective controls were starved overnight in IMDM GlutaMAX without FCS. The next day, 2.5 × 10^5^ cells were added to the upper side of the Boyden chamber (Cell Biolabs, Inc., San Diego, CA USA) in triplicate in serum-free medium. FBS-conditioned medium was added to the lower chambers. After 24 h of incubation at 37 °C, non-migrated cells were carefully removed from the upper side of the chamber. Cells that had migrated to the bottom side of the chamber were fixed, and stained with crystal violet and destained. The OD^560^ was measured on a Tecan Infinite M200 Pro spectrophotometer (Tecan, Männedorf, Switzerland).

### 2.7. Clonogenic Assay

Serial dilutions of cells were prepared starting from a total of 5 × 10^4^ until achieving a dilution of eight cells/well of a six-well multi-well plate. Cells were seeded and maintained in complete medium. After eight days, colonies were gently washed with PBS, fixed in methanol and stained with 0.05% (*w*/*v*) crystal violet diluted in 25% methanol. Stained plates were scanned and enlarged printouts used to count colonies (a colony is defined as a group of at least 50 cells or more). The most significant dilution of cells (0.2 × 10^3^) is displayed.

### 2.8. Anchorage-Independent Growth Assay

Stably transfected cells were treated as described by Piazzi et al. [17]. Briefly, cells were mixed with complete cell culture medium containing 0.6% methyl cellulose and plated over a layer of solidified complete cell culture medium containing 0.8% agar in six-well culture plates at a density of 2 × 10^4^ cells per plate. Plates were incubated under standard growth conditions. The medium was replenished every 3–4 days. After one or two weeks, depending on the cell type, colonies were observed and photographed under a light microscopy (Carl Zeiss, Munich, Germany). Whereas cellular aggregates were omitted from the analysis, the diameter of regularly shaped colonies (spheroids) was measured using AxioVision software (AxioVision, Feldbach, Switzerland).

### 2.9. Protein Extracts and Immunoblot

Cells were trypsinized, collected by centrifugation and lysed in SDS-lysis buffer (20 mM Tris-HCl pH 7.5, 1% SDS, 5% 2-Mercaptoethanol). Total lysate (30–80 ug) was resolved by SDS-PAGE, electro-transferred onto nitrocellulose membranes (Santa Cruz Biotechnology, DBA Italia SRL, Segrate, Italy) and immunoblotted with the indicated antibodies. Antibodies used were: anti-Akt (1:500), anti-actin (1:1000), anti-MMP2 (1:500), anti-p53 (1:200) and anti-Lamin A/C (1:200), all from Santa Cruz Biotechnology; anti-p-Akt2(Ser474) (1:1000) and anti-Akt2 (1:1000) from Cell Signaling (Cell Signaling Technology, EuroClone, Pero, Italy); rabbit polyclonal anti-Ankrd2 (1:2000) (PtgLab, Proteintech Rosemont IL, USA), anti-GAPDH (1:8000) and anti-MMP9 (1:1000), from Merck (Merck-Millipore, Darmstadt, Germany); and anti-Flag (1:1000) and anti-β-tubulin (1:2000) (both from Sigma-Aldrich, St. Louis, MO, USA). HRP-conjugated secondary antibodies were from Santa Cruz Biotechnology or Abcam (Abcam, Cambridge, UK). Oligoclonal anti-phospho-Ankrd2(Ser99) was developed by immunizing rabbits with the peptide CGQERVRKT(pS)LDLRRE (residues 91–105 of the S-Ankrd2 sequence) coupled with maleimide-activated keyhole limpet hemocyanine (KLH) through the N-terminal cysteine. Rabbits were injected four times at three-week intervals, with 0.5 mg of peptide-protein conjugates emulsified with Freund’s adjuvant (1:1 *v*/*v*). Antiserum was purified using an immobilized peptide affinity resin (Sulfo Link Coupling Gel, Pierce, Monza, Italy) according to manufacturer’s instructions. The immunoglobulin fraction was obtained from sera by Protein A—agarose chromatography. Before being used, antibody specificity was evaluated on HEK-293T cells transfected with plasmids encoding Ankrd2wt and its mutant forms on S99, that is the unphosphorylatable Ankrd2(S99A), and the phospho-mimetic one, Ankrd2(S99E) (Figure S1).

### 2.10. Immunofluorescence

Cells were seeded on coverslips, and once they reached 80% confluence, they were fixed in methanol for 7 min at room temperature. Coverslips were then saturated with 5% BSA and incubated with polyclonal anti-Ankrd2 (Ptglab, Proteintech, 1:100) overnight at 4 °C, or with monoclonal anti-Flag (Sigma-Aldrich, 1:500) for 1 h at room temperature. Nuclei were stained with DAPI. Cells were finally observed with a Nikon Eclipse Ni epifluorescence microscope (Nikon Healthcare, Tokyo, Japan), and images were captured with NIS-Elements 4.3 AR software (Nikon Healthcare, Tokyo, Japan).

### 2.11. Gelatin Zymography

A total of 4 × 10^5^ cells from OS clones were plated in 35 mm petri dishes. The next day, the medium was replaced with a serum-free media for 36 h. Media was collected and pelleted and proteins were quantified. Gelatinase activity was determined under non-reducing conditions on gelatin co-polymerized 9% SDS-PAGE gels. After the run, gels were washed three times in washing buffer (2.5% Triton X-100, 50 mM Tris-HCl pH 7.5, 5 mM CaCl_2_ and 1 μM ZnCl_2_) and finally incubated in a solution containing 50 mM Tris-HCl pH 7.5, 5 mM CaCl_2_ and 1 μM ZnCl_2_, at 37 °C overnight. After staining with Coomassie brilliant blue R250 (Bio-Rad Laboratories, Hercules, CA, USA), gels were destained in water and the activity of MMP2 and MMP9 was evaluated by densitometric analysis of unstained bands with ImageJ software, Bethesda, MD, USA.

### 2.12. Image Processing and Statistical Analysis

All the images shown are representative of at least three independent experiments carried-out under the same conditions. Images from immunochemical and immunofluorescence studies were processed using Photoshop CS4 (Adobe Systems, Inc., San Jose, CA, USA). Densitometric analysis was performed by ImageJ (National Institute of Health, Bethesda, MD, USA). Data are expressed as the mean ± SD of the number of the indicated biological replicates. Data were analyzed by Student’s *t*-test. *p* < 0.01 was accepted as significant.

## 3. Results

### 3.1. Ankrd2 is Expressed in Human Osteosarcoma Cell Lines

**Figure 1:**
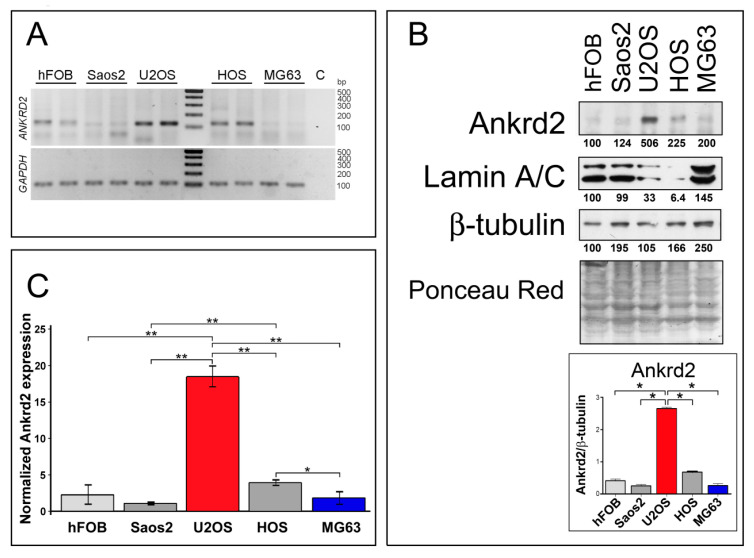
Ankrd2 is expressed in cell lines derived from human osteosarcoma and human normal osteoblasts. (**A**) Detection of the *ANKRD2* transcript in cell lines obtained from human normal osteoblasts (hFOB) and from human osteosarcoma (Saos2, U2OS, HOS and MG63). The fragment was amplified by RT-PCR using cDNA from at least three passages of tumor cells. Housekeeping *GAPDH* was used as a quality control. No cDNA was added to the control reaction (**C**). Length of the amplicons are presented in Table S1. (**B**) Total cell lysates from hFOB, Saos2, U2OS, HOS and MG63 were resolved by SDS-PAGE and assayed for Ankrd2 expression. A positive signal was observed at 38 kDa corresponding to the expected molecular weight. Levels of lamin A/C were also determined. β-tubulin and ponceau-red staining of the filter were used as equal loading controls. Expression of Ankrd2 is presented as the ratio between the values of densitometric analysis of the bands of anti-Ankrd2 against those of anti-β-tubulin. Blots are representatives of four repetition. (**C**) Relative quantification of *ANKRD2* mRNA expression in hFOB, Saos2, U2OS, HOS and MG63 cell lines as determined by qPCR. *GAPDH* served as the internal reference. The data are expressed as 2^−ΔCt^ × 10^5^ and presented as mean ± SD of at least three biological replicates. Statistical analysis was performed with a paired two-tail Student’s *t*-test. * *p* < 0.01, ** *p* < 0.001. Average Ct ± SD values are presented in Table S2.

To gain more insight into Ankrd2 involvement in osteosarcoma oncogenesis and progression, Ankrd2 expression levels were first assessed in cell lines derived from human OS. In particular, Saos2, U2OS, HOS and MG63 cells were chosen as representative models to study this malignancy. As a control, Ankrd2 expression was also monitored in the normal human osteoblast cell line, hFOB. A non-quantitative analysis of total RNAs (Figure 1A) revealed that the Ankrd2 transcript is expressed to detectable levels in all tested cell lines, including the control hFOB cells. A protein expression analysis (Figure 1B) revealed that the level of Ankrd2 protein varied between the cell lines. U2OS expressed the most Ankrd2 followed by HOS. Control osteoblasts (hFOB) and the Saos2 and MG63 OS cell lines expressed the least Ankrd2 (Figure 1B). Immunoblot results were confirmed by a quantitative PCR (qPCR) analysis of the total transcripts obtained from hFOB, Saos2, U2OS, HOS and MG63 cultures (Figure 1C). Interestingly, Figure 1B also highlights the observation that lamin A/C protein expression negatively correlated with that of Ankrd2. Lamin A/C is the major constituent of the nuclear lamina and has been associated with the nuclear translocation of Ankrd2 [18]. Previously, we have demonstrated that an impaired nuclear lamina, due to mutations of the gene encoding lamin A/C, i.e., *LMNA*, is responsible for an unusual nuclear recruitment of Ankrd2 even under basal conditions [18]. Of note, lamin A/C has been recently correlated with the migration potential of OS cells [19]. The subcellular localization of Ankd2 in the OS cell lines was next evaluated by immunofluorescence. In accordance with the immunoblot results, the expression of Ankrd2 was almost undetectable in hFOB, Saos2 and MG63 cells (not shown), whereas in U2OS, as well as in HOS cells, Ankrd2 was detected in the cytoplasm, in the nucleoplasm and saddled to the nuclear rim (Figure S2).

### 3.2. Characterization of Clones of OS Cell Lines Stably Expressing wt and S99A Ankrd2

**Figure 2:**
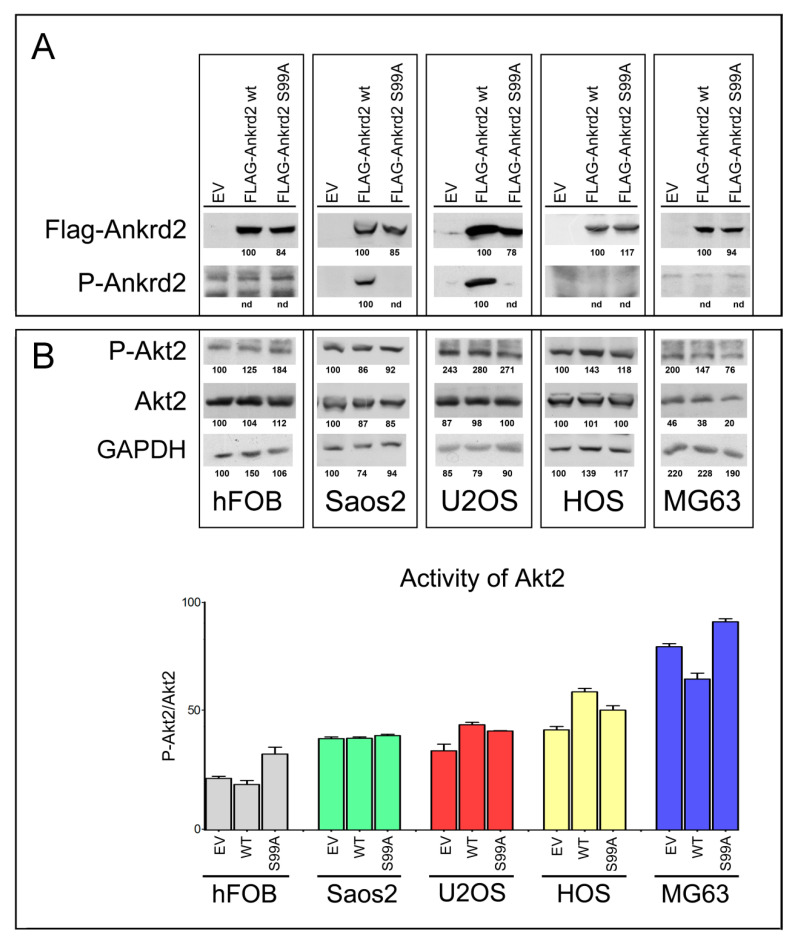
Characterization of OS cell lines stably expressing Ankrd2 wt and mutant S99A. (**A**) Total cell lysates obtained from clones of hFOB, Saos2, U2OS, HOS and MG63 overexpressing Flag-tagged Ankrd2 forms (wt and S99A), or transfected with empty vector (EV), were assayed for the expression of ectopic Ankrd2, by the use of an antibody against its Flag-tag, as well as the phosphorylation profile of its S99 [P-Ankrd2, phospho-Ankrd2 (Ser99)]. (**B**) Fifty μg of total lysates from the same samples of A) were analyzed with the indicated antibodies [P-Akt2, phospho-Akt2 (Ser474)]. The activation status of Akt2 is presented as the ratio between the values of densitometric analysis of the bands of anti-p-Akt2 against those of anti-Akt2. Blots represent the best of four repetitions.

To evaluate the involvement of Ankrd2 in OS progression, Saos2, U2OS, HOS and MG63 OS cell lines and control hFOB cells were stably transfected with an expression vector encoding Flag-tagged Ankrd2wt (Flag-Ankrd2wt). Moreover, since in C2C12 myoblasts most of the cellular functions of Ankrd2 are mediated by its phosphorylation on S99 by Akt2 [10,11], and since Akt2 plays a key role in the progression of several types of cancer including OS [20], we were also interested in determining if S99 might be involved in oncogenesis of OS. For these reasons, cells were also transfected with a mutant form of Flag-tagged Ankrd2, which cannot be phosphorylated on S99, Ankrd2(S99A) (Flag-Ankrd2(S99A). The results in Figure 2A demonstrate that both Ankrd2 forms (wt and S99A) were expressed in all cell lines. Using a custom made phospho-specific antibody, the level of Ankrd2 phosphorylated on S99 was also analyzed. Figure 2A shows that in U2OS and Saos2 clones, ectopic Flag-Ankrd2wt was phosphorylated, whereas the phosphorylation control Flag-Ankrd2(S99A) was not. In hFOB, HOS and MG63 clones, phosphorylation of Flag-Ankrd2wt on S99 was not detected (Figure 2A). To test the hypothesis that the lack of Ankrd2 phosphorylation on S99 in hFOB, HOS and MG63 clones expressing Flag-Ankrd2wt is due to altered Akt2 activity compared to U2OS and Saos2, the activation status of Akt2 in these cells was assayed by western blot, using a phospho-specific antibody (Figure 2B). The expression and activation of Akt2 in hFOB, HOS and MG63 cells was comparable to that in U2OS and Saos2 cell lines, indicating that unlike myocytes [10], Akt2 activation and phosphorylation of Ankrd2 on S99 do not play a consistent role in osteosarcoma cell lines.

Cellular distribution of ectopically expressed Flag-Ankrd2wt in OS cells was next assayed by immunofluorescence analysis (Figure S3). In Ankrd2-expressing hFOB, HOS and MG63 clones, Flag-Ankrd2wt had a distinctive cytoplasmic localization. In contrast, in Saos2 and U2OS clones, Flag-Ankrd2wt had a slight nuclear as well as cytoplasmic distribution (Figure S3).

### 3.3. Effect of Ankrd2 Overexpression on the Proliferation of OS Cells

**Figure 3:**
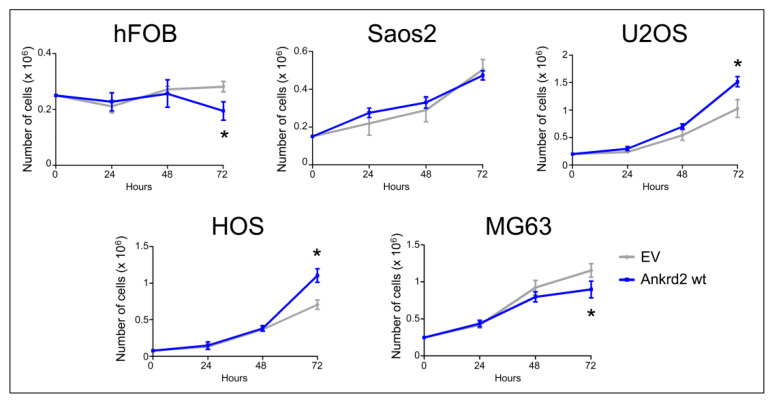
Ankrd2 overexpression affects proliferation of cell lines from OS and normal osteoblasts. Stable clones of hFOB, Saos2, U2OS, HOS and MG63 overexpressing Flag-Ankrd2wt or transfected with empty vector (EV) were seeded as indicated in six-well tissue culture plates. After 24, 48 and 72 h from seeding, cells were individually collected by trypsinization, stained with trypan blue and living cells counted under light microscopy using a hemocytometer. * *p* < 0.05, vs. control (EV). Data are representative of a minimum of five independent experiments. Data were analyzed and graphed using the GraphPad Prism software.

The contribution of Ankrd2 to proliferation of OS cells was tested in Flag-Ankrd2wt overexpressing hFOB, Saos2, U2OS, HOS and MG63 cells. The growth rate and viability were measured for three consecutive days. Compared to the respective control (empty vector-transfected), Flag-Ankrd2wt expression promoted cell proliferation in U2OS and HOS cells, whereas its overexpression induced a modest, although significant reduction in proliferation of MG63 and hFOB cells compared to the respective control (empty vector) (Figure 3). Overexpression of Flag-Ankrd2 had no effect on the proliferation of Saos2 cells (Figure 3). It is interesting to note that the difference in proliferation observed upon the overexpression of Ankrd2 might be correlated with the level of expression of lamin A/C, which is involved in the nuclear shuttling of Ankrd2 [18].

### 3.4. Effect of Ankrd2 Overexpression on OS Cellular Motility

Malignant transformation, besides the acquisition of novel phenotypic properties, including unlimited proliferative potential, reduced growth factor requirements, or loss of specialized cell functions, is also characterized by the ability of cells to overcome contact inhibition and to grow in a semisolid environment. In order to evaluate the involvement of Ankrd2 in cell motility and establish a role for Ankrd2 in metastases production and spreading, Flag-Ankrd2wt-expressing clones were subjected to a series of standard tests for functional analyses, including scratch test, migration and anchorage-independent and -dependent growth assays.

#### 3.4.1. Wound Healing

**Figure 4:**
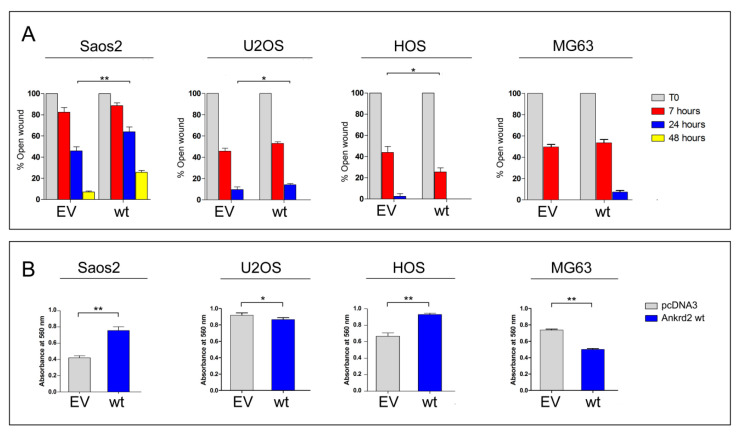
Ankrd2 overexpression affects the motility of human OS cell lines. (**A**) The 90%-confluent monolayers of OS clones overexpressing Flag-Ankrd2wt (wt) or transfected with empty vector (EV) were wounded with a 200 μL pipette tip. Three “wounds” were formed on each culture dish in duplicate per experiment. Images were acquired by light microscopy at the time of the scratch (T0) and after 7, 24 and, where indicated, 48 h. Measures of the cell free area were taken by the AxioVision software, Feldbach, Switzerland. Data were plotted as the percentage of open wound compared to its original size, at T0, and analyzed using the GraphPad Prism software. Statistical analysis was performed with a paired two-tail Student’s *t* test. * *p* < 0.05, ** *p* < 0.01. (**B**) A total of 3 × 10^4^ cells of Saos2, U2OS, HOS and MG63 clones overexpressing Flag-Ankrd2wt (wt) or those that were transfected with empty vector (EV), were loaded into the upper Boyden chamber in serum-free media and assayed for chemotactic potential. After 5 h of culture in the presence of chemo-attractant, cells were stained with crystal violet and the absorbance at 560 nm was measured. Data were analyzed with the GraphPad Prism software. Data are representative of two independent experiments. Statistical analysis was performed using an unpaired two-tail Student’s *t* test. *p* < 0.01.

To test the contribution of Ankrd2 to the motility of Saos2, U2OS, HOS and MG63 cells, we performed a scratch-mediated wound healing assay on the corresponding Flag-Ankrd2-expressing clones. Of note, hFOB clones were not subjected to this assay because of their propensity to differentiate when reaching confluence. Results in Figure 4A show that under standard growing conditions, 7 h after the scratch, the expression of Flag-Ankrd2wt in Saos2 did not induce any variation in the rate of healing compared to control. Interestingly, 24 h after the scratch, the expression of Flag-Ankrd2wt reduced the healing rate shown by control cells by about 37% (Figure 4A). Likewise, in U2OS cells, the expression of Flag-Ankrd2wt induced no significant variation in the rate of wound healing with respect to the control 7 h after wounding (Figure 4A). After 24 h, the rate of wound healing in the U2OS cells expressing Flag-Ankrd2wt as compared to control cells was reduced while 48 h after scratching, the wound was completely healed in both control and Flag-Ankrd2wt-expressing cells (Figure 4A). In HOS cells, 7 h after the scratch, the expression of Flag-Ankrd2wt induced an increase in the rate of wound closure of around 35% with respect to the control (Figure 4A). After 24 h, Flag-Ankrd2wt expressing HOS cells were perfectly healed (Figure 4A). In MG63 expressing Flag-Ankrd2wt, the width of the scratch was similar to control after 7 h. Interestingly, 24 h after the scratch, the wound of Ankrd2wt-expressing MG63 cells was still open (Figure 4A), although this might also be a consequence of the drop of cell proliferation induced by Ankrd2wt expression (Figure 3). Overall, these data suggest that Ankrd2 contributes to cell motility in a cell line-dependent fashion.

#### 3.4.2. Chemotactic Migration

To assess cell migration toward a chemo-attractant (chemotaxis), Ankrd2wt-expressing Saos2, U2OS, HOS and MG63 cells were subjected to a Boyden chamber transwell migration assay. Cells that had been serum starved overnight were plated in the upper side of the Boyden chamber surrounded by medium supplemented with serum. Of note, due to the low tolerance to starving conditions, it was not possible to perform the assay with hFOB. After 24 h, the chemotactic-dependent motility of cells versus the chemo-attractant (serum) was tested. The results shown in Figure 4B revealed that in Saos2 and HOS cells, the ectopic expression of Flag-Ankrd2wt resulted in an increase in cell migration compared to empty vector-transfected counterparts (Figure 4B). In contrast, in U2OS and MG63 cells, the ectopic expression of Flag-Ankrd2wt impaired cell migration (Figure 4B). In HOS cells, the expression of both the Ankrd2 forms induced an increase of cell migration (Figure 4B). Overall, these data demonstrate that, albeit in a cell line-dependent manner, Ankrd2 expression can influence the chemotactic-dependent migration of OS cells.

#### 3.4.3. Clonogenic Potential

**Figure 5:**
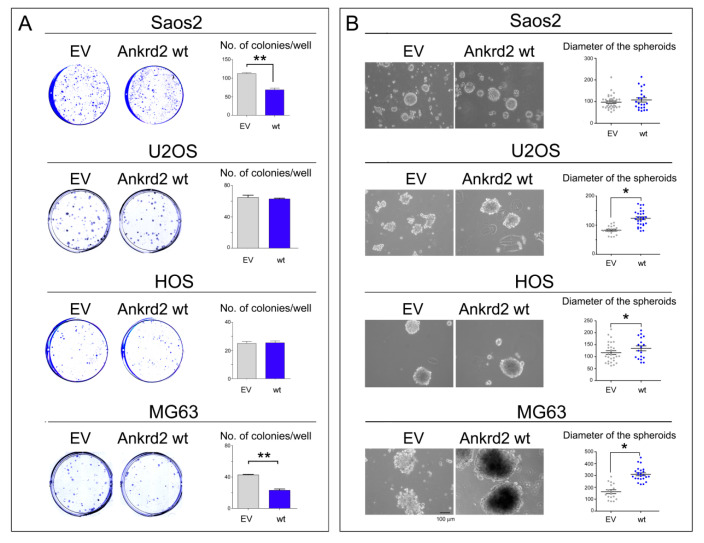
Ankrd2 overexpression affects clonogenicity of OS cell lines. (**A**) 1.2 × 10^2^ cells from Saos2, U2OS, HOS and MG63 clones overexpressing Flag-Ankrd2wt, or that were transfected with empty vector (EV), were plated in multi-well plates and allowed to grow for two weeks. Colonies with >50 cells were counted and graphed using the GraphPad Prism software. Statistical analysis was performed using an unpaired two-tail Student’s *t* test. ** *p* < 0.001. (**B**) Saos2, U2OS, HOS and MG63 clones were seeded at a density of 2 × 10^5^ cells/35 cm^2^ culture dish in complete media containing methyl-cellulose onto a layer of 0.8% agar and allowed to grow. Spheroids were observed under light microscopy, and the diameter of spheroids (reported in μm) composed of >50 cells were measured by AxioVision software and graphed into a vertical scatter plot using the GraphPad Prism software. The bar is 100 μm. Statistical analysis was performed with a two-tail Student’s *t*-test. * *p* < 0.01.

To test the ability of Ankrd2 to drive colony formation from a single cell, Ankrd2-expressing OS clones were subjected to colony formation assays (CFA). Serial dilutions of Flag-Ankrd2-expressing OS cells were seeded and allowed to grow for one week or until colonies with >50 cells were formed. In U2OS and HOS cells, overexpressed Flag-Ankrd2wt was unable to enhance the clonogenic potential in comparison to control cells (Figure 5A). On the contrary, the clonogenic potential of parental Saos2 and MG63 cells was reduced by ectopic expression of Flag-Ankrd2wt, (Figure 5A). Clonogenic ability was not observed for hFOB cells transfected with empty vector or expressing Flag-Ankrd2wt (not shown).

#### 3.4.4. Anchorage Independent Growth

To evaluate the ability of Ankrd2 to affect the anchorage-independent growth of hFOB, Saos2, U2OS, HOS and MG63 cell lines, Ankrd2-expressing clones were seeded and grown in media containing methyl cellulose which prohibits cellular attachment. Cells were cultured until three-dimensional (3D) spheroids made of >50 cells were observed and their diameter was measured. The results shown in Figure 5B demonstrated that the ectopic expression of Ankrd2 was able to speed the formation of regularly-shaped spheroids in OS cells in all the cell lines tested, except for Saos2, in which the ability of control cells was not modified by the overexpression of Ankrd2. On the other hand, neither parental nor Ankrd2-expressing hFOB clones were able to form spheroids (not shown). Overall, these results indicate that Ankrd2 might be involved in the modulation of invasiveness and migratory potential of OS cells.

### 3.5. Effect of Ankrd2 Overexpression on Degradation of the Extracellular Matrix

**Figure 6:**
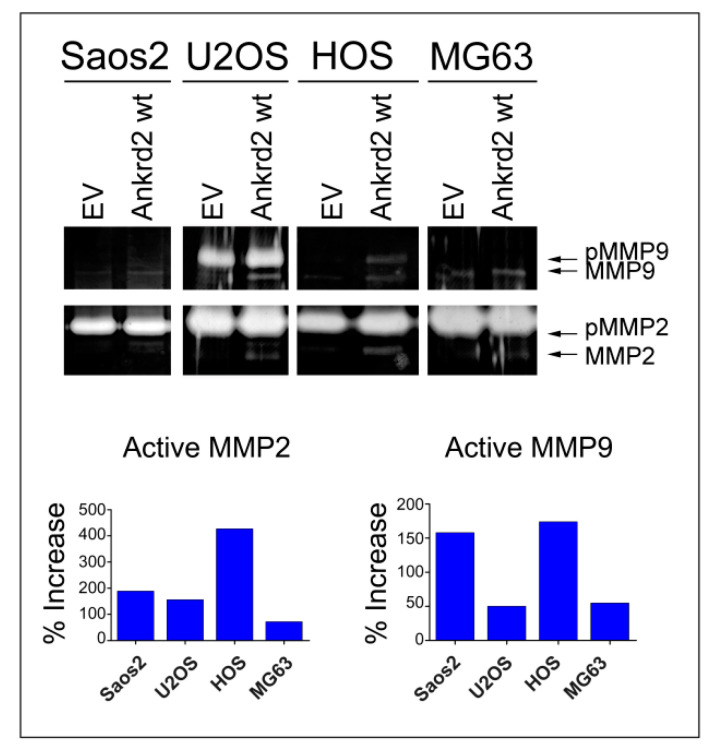
Ankrd2 overexpression affects the activity of MMP2 and MMP9. A total of 1 × 10^6^ cells from Saos2, U2OS, HOS and MG63 clones overexpressing Flag-Ankrd2wt, or transfected with empty vector (EV), were seeded into a 35 cm^2^ petri dish and serum starved for 24 h. After additional 36 h, media were collected and assayed for their gelatinolytic activity. Total protein (7 μg) from media was resolved on a gelatin embedded polyacrylamide gel under non denaturing conditions. Gelatinolytic activity of MMP2 and MMP9 was visualized as unstained (white) bands. Protease activity of MMP2 and MMP9 in OS clones expressing Ankrd2 wt is reported as the percentage increase with respect to control (empty vector-transfected) clones. MMP2 and MMP9 appeared as two bands corresponding to the inactive pro-enzyme (indicated as pMMP2 and pMMP9) and to the cleaved active form (MMP2 and MMP9).

Matrix metalloproteinases (MMPs) play a key role in cancer cell migration and invasion of surrounding tissues, by digesting the extracellular matrix (ECM) and the basement membranes. Since MMP2 and MMP9 are two of the most characterized MMPs in cancer metastasis [21], and since Ankrd2 modulates cell motility, we tested the possibility that Ankrd2 might promote invasion by modulating the activity of these MMPs. Confluent Flag-Ankrd2-expressing OS cells were serum starved. After 36 h, media were processed for the evaluation of gelatinolytic activity. Results shown in Figure 6 demonstrated that in all the cell lines tested, the overexpression of Flag-Ankrd2wt increased MMP2 and MMP9 activity.

### 3.6. Effect of Ankrd2 Silencing in OS Cells

**Figure 7:**
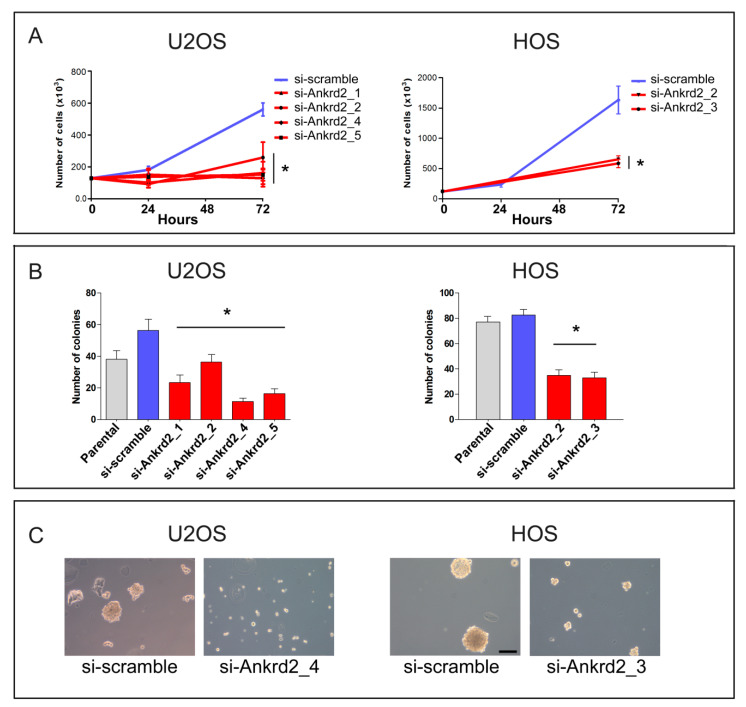
Silencing of Ankrd2 expression reduces proliferation and clonogenic potential of U2OS and HOS cells. (**A**) Stable clones of Ankrd2-silenced U2OS and HOS cells were seeded at a density of 2 × 10^5^ cells/well in six-well tissue culture plates. Then, 24, 48 and 72 h after seeding, cells were individually collected and counted under light microscope. As control, the proliferation rate of respective parental and si-scramble cells was also determined. * *p* < 0.01, vs. si-scramble. Data are representative of a minimum of three independent experiments. Data were analyzed and graphed using the GraphPad Prism software, San Diego, CA, USA. (**B**) The same clones as in (**A**) were plated at a density of 13.3 cells/cm^2^ and allowed to grow for two weeks. Colonies with >50 cells were counted and the results were graphed using the GraphPad Prism software. Statistical analysis was performed using an unpaired two-tail Student’s *t*-test. * *p* < 0.01 vs. si-scramble. (**C**) Control (si-scramble) and Ankrd2 silenced clones were seeded at a density of 2 × 10^5^ cells/35 cm^2^ culture dish in complete media containing methyl-cellulose on a 8% layer of 0.8% agar and allowed to grow. Spheroids were observed by light microscopy. Images of U2OS (clone 4, si-Ankrd2_4) and HOS (clone 3, si-Ankrd2_3) unable to form spheroids when partially depleted of Ankrd2 are shown. The bar is 100 μm.

To further decipher the role of Ankrd2 in tumor progression, OS cell lines expressing the higher Ankrd2 amount, i.e., U2OS and HOS cells, were next subjected to Ankrd2 silencing by stable transfection with Ankrd2 specific siRNA. After one month of selection, the obtained clones were evaluated for Ankrd2 expression (shown in Figure S4) and those expressing the lowest amount of Ankrd2 were assayed for proliferation, as well as attachment-dependent and -independent growth assays. The results presented in Figure 7A demonstrated that in U2OS and HOS cells, the knock-down of Ankrd2 significantly reduced the proliferation rate compared to si-scramble and parental counterparts (Figure 7A). When assayed for their ability to grow under anchorage-dependent conditions, all the Ankrd2-silenced clones formed fewer and smaller colonies compared to respective si-scramble or parental counterparts (Figure 7B). Furthermore, when cultured under attachment-independent conditions, the ability of Ankrd2-silenced clones to form spheres was reduced or completely abrogated with respect to controls (most significative images are shown in Figure 7C).

## 4. Discussion

Prior to this study, Ankrd2 had been essentially investigated in muscle cells, where multiple reports suggested its involvement during muscle differentiation under both physiological and stress conditions [6,10,18] as well as a role as a downstream signal transducer in mechanosignaling pathways in mature muscle cells [7]. Other studies have in parallel described an increased expression of Ankrd2 in cells and tissues derived from some primary tumors, such as rhabdomyosarcoma and renal oncocytoma [3,16,22]. Immunohistochemical data, collected by the Human Protein Atlas also report an increase of Ankrd2 expression in tissue from colorectal cancer, breast cancer, prostate cancer and lung carcinoma (https://www.proteinatlas.org/). An increase in the Ankrd2 transcript in osteosarcoma-derived cell lines has also been described by a transcriptomic data set published on EMBL Expression Atlas (https://www.ebi.ac.uk/gxa/home). Moreover, an emerging role for Ankrd2-homolog Ankrd1 in cancer development has been proposed. A remarkable number of papers have demonstrated that Ankrd1 is involved in the progression of cancers such as those afflicting ovaries [23], breast [24], pancreas [25] and in the chemoresistance of lung cancer [26].

In the study presented here, we report on Ankrd2 basal expression in cell lines derived from human osteosarcoma and demonstrate for the first time that the modulation of the level of expression of Ankrd2 affects certain oncogenic properties of these cells. Our results, summarized in Table 1, show that the role of Ankrd2 in osteosarcoma progression is heterogeneous and, in some cases, particular to the cell line tested, suggesting that cell line-specific factors might contribute to the overall Ankrd2-related effects. Nonetheless, we have also observed that Ankrd2 silencing had a negative impact on cancer cells progression (Figure 7). Altogether, these findings allowed us to suppose that Ankrd2 has a permissive role in cancer progression. However, according to our hypothesis, Ankrd2 might not be a master regulator of osteosarcoma progression, but rather one of the last effectors of one (or more) signaling cascade(s) that in the cell lines differently regulate Ankrd2 activity through post translational modifications (PTMs), localization or stability. In this context, the ectopic expression of Ankrd2 might thus result in slightly differing cellular effects. On the contrary, the depletion of Ankrd2, by blocking the final downstream effects of multiple signaling cascades, might impair in a more unambiguous way the fate of osteosarcoma cells.

The findings on the permissive role of Ankrd2 in spheroid formation (Figure 5B) and in the modulation of MMP2 and MMP9 activities (Figure 6), as well as the evidence that Ankrd2-depleted clones lose the ability to form spheroids (Figure 7C), hint at a positive role for Ankrd2 in triggering metastases and tumor spreading. Therefore, considering the mechano-transducer role previously assigned to Ankrd2 [6], in our models, Ankrd2 might transduce signals in response to mechanical and stress stimuli elicited by inter- and intra-cellular mechanical forces.

In a previous study, we identified the phosphorylation of Ankrd2 S99 by Akt2 as an important mediator of Ankrd2 functions upon oxidative stress [10,11]. In an attempt to evaluate if the modulation of S99 phosphorylation might be involved in the regulation of the events observed in Ankrd2 overexpressing OS-derived cell lines, and may, thus, be exploited to reduce cancer progression, all the biochemical and functional assays performed with wild-type Ankrd2 were contextually performed with the unphosphorylatable mutant form of Ankrd2, i.e., Ankrd2(S99A) (Figure 2 and data not shown). However, the results obtained were not consistent, probably due to the existence of several cell type-specific regulatory mechanisms modulating PTM(s) of Ankrd2 on S99 and other sites. Due to the absence of a clear reversion of the phenotype in cells expressing Ankrd2(S99A) mutant, we assume that S99 is not essential for Ankrd2 functions related to tumor progression, and thus, cannot be considered a good therapeutic target to counteract cancer growth.

A recent study performed in head and neck squamous cell carcinoma (HNSCC) has presented Ankrd2 as a negative regulator of the progression of this cancer [27]. According to the authors, Ankrd2 is involved in the block of cancer cell proliferation by directly inhibiting the NF-κB signaling pathway. The opposing effects exhibited by Ankrd2 in OS cells and in HNSCC [27] hint at a role of Ankrd2 as a “double-faced” cancer gene, a well-documented peculiarity of certain genes exhibiting oncogenic or tumor-suppressor behavior depending on the biological context [28,29]. Interestingly, a homolog of Ankrd2, Ankrd23 [1] was recently identified as a potential dual-role cancer driver gene acting as an oncogene in renal clear-cell carcinoma, and a tumor suppressor in bladder urothelial carcinoma [30].

Further evidence, reported in the Supplementary Materials in Figure S5, revealed that Ankrd2 is expressed in cell lines derived from human rhabdomyosarcoma (RMS), albeit with a different profile of expression (Figure S5). Our data reports that the ectopic expression of Ankrd2 in one of these cell lines does not affect proliferation or attachment-independent growth of parental cells, while it minimally impairs attachment-dependent growth (Figure S5). Hopefully, a thorough investigation of the Ankrd2 interactome in different types of cancers will help us to explain the controversial role of Ankrd2 in cancer progression.

In the future, the evaluation of the Ankrd2 expression level in cells from a cohort of primary and metastatic OS will assist in ascertaining if Ankrd2 basal expression in OS correlates with tumor grade and severity of the disease. Moreover, since Ankrd2 is able to modulate pro-inflammatory signaling pathways, including NF-κB, studying the relation between Ankrd2 and the microenvironment of OS might open a new chapter on how Ankrd2-dependent mechanotransduction might influence the progression and metastases formation of this disease. To this regard, Wagner et al. have described the generation of an innovative humanized tissue-engineered bone organ (hTEBO) for preclinical research on primary bone tumors. Containing human bone matrix and marrow components in one organ, this model reproduces a bone microenvironment fully mimicking human physiology [31,32,33]. By the injection of either Ankrd2- or mock-transfected OS cell lines into these preclinical experimental models, we might be able in the future to investigate the effects of Ankrd2 expression on both the progression and microenviroment composition of OS. These points, together with the possibility to reduce Ankrd2 expression in vivo, will shed a light on novel therapeutic perspectives.

## 5. Conclusions

Despite being detected in tumor cells and tumor tissues, any role of Ankrd2 in OS development, maintenance and progression has not been described so far ever described any role of Ankrd2 in OS development, maintenance and progression. The main aim of this study was to demonstrate the involvement of Ankrd2, a protein known for being essentially expressed in muscle, in OS patho-physiology, by exploiting two- and three-dimensional cultures from OS cell lines stably expressing this protein. Our data show that Ankrd2 is able to impact the proliferation, motility and clonogenic potential of OS cell lines. Further and more in-depth studies aimed at determining the intracellular molecular cascades ruled by Ankrd2, its interactome, its PTMs and regulation of its expression, and how all these come into play, are needed to take full advantage of this protein as a potential therapeutic target.

## Figures and Tables

**Table 1:** Effects of the ectopic expression of Ankrd2 on biological functions of Ankrd2-expressing OS cell lines. Positive (+), negative (-) or not-statistically significant (=) contributions of Flag-Ankrd2 on cellular functions of Saos2, U2OS, HOS and MG63 cell lines have been reported with respect to their own parental (empty vector-transfected) counterpart.

Osteosarcoma-Derived Cell Line	Effect on Proliferation	Effect on Migration (24 h from Wounding)	Effect on Transwell Migration	Effect on Clonogenicity (Anchorage Dependent)	Effect on Clonogenicity (Anchorage Independent)	Effect on MMP2/9 Activity
**Saos2**	=	-	+	-	=	+
**U2OS**	+	-	-	=	+	+
**HOS**	+	+	+	=	+	+
**MG63**	-	-	-	-	+	+

## Data Availability

Data is contained within the article or supplementary material.

## Supplementary Materials

The following are available online at https://www.mdpi.com/2072-6694/13/2/174/s1, Figure S1: Validation of the efficacy of the anti Ankrd2-phospho-Ser99 antibody, Figure S1B: Original uncropped blots of Figure 1B; Figure S2: Immunofluorescence analysis of endogenous Ankrd2 in U2OS and HOS cell lines; Figure S2A: Original uncropped blots of Figure 2A; Figure S2B: Original uncropped blots of Figure 2B; Figure S3: Analysis of subcellular distribution of ectopically expressed Ankrd2wt in Ankrd2-overexpressing clones from hFOB, Saos2, U2OS, HOS and MG63 cell lines, Figure S4: Analysis of Ankrd2 expression level in clones of U2OS and HOS cells stably expressing a Si-Ankrd2 transcript, Figure S5: Effects of ectopic expression of Ankrd2 in a cell line derived from human rhabdomyosarcoma, Table S1: List of primers used for RT-PCR and qPCR amplification of Ankrd2 and GAPDH fragments, Table S2: Average Ct ± SD values for *ANKRD2* and *GAPDH* genes in cell lines derived from human osteoblasts (hFOB) and human OS.
