# Supplementary material for: Ectopic Expression of Ankrd2 Affects Proliferation, Motility and Clonogenic Potential of Human Osteosarcoma Cells
Source: Cancers (Basel). 2021 Jan 6;13(2):174. doi: 10.3390/cancers13020174 (PMC7825408; doi:10.3390/cancers13020174)
Supplement: Supplementary file 1 [file cancers-13-00174-s001.zip › Supplementary files/Rev_Piazzi et al_Table S2.docx]

|  | ***ANKRD2*** | | ***GAPDH*** | |
| --- | --- | --- | --- | --- |
|  | avg Ct | SD | avg Ct | SD |
| **hFOB** | 29.919 | 0.851 | 14.436 | 0.216 |
| **Saos2** | 31.118 | 0.341 | 14.658 | 0.062 |
| **U2OS** | 26.886 | 0.542 | 14.483 | 0.151 |
| **HOS** | 29.269 | 0.359 | 14.644 | 0.179 |
| **MG63** | 30.209 | 0.733 | 14.320 | 0.109 |

Supplemental Table S2
