# Supplementary material for: Ectopic Expression of Ankrd2 Affects Proliferation, Motility and Clonogenic Potential of Human Osteosarcoma Cells
Source: Cancers (Basel). 2021 Jan 6;13(2):174. doi: 10.3390/cancers13020174 (PMC7825408; doi:10.3390/cancers13020174)
Supplement: Supplementary file 1 [file cancers-13-00174-s001.zip › Supplementary files/List of Supplementary files.docx]

**Figure Legends of Supplementary Files**

**Figure S1**: Validation of the efficacy of the anti Ankrd2-phospho-Ser99 antibody;

**Figure S1B:** Original uncropped blots of Figure 1B;

**Figure S2**: Immunofluorescence analysis of endogenous Ankrd2 in U2OS and HOS cell lines;

**Figure S2A:** Original uncropped blots of Figure 2A;

**Figure S2B:** Original uncropped blots of Figure 2B;

**Figure S3**: Analysis of subcellular distribution of ectopically expressed Ankrd2wt in Ankrd2-overexpressing clones from hFOB, Saos2, U2OS, HOS and MG63 cell lines;

**Figure S4**: Analysis of Ankrd2 expression level in clones of U2OS and HOS cells stably expressing a Si-Ankrd2 transcript;

**Figure S5**: Effects of ectopic expression of Ankrd2 in a cell line derived from human rhabdomyosarcoma;

**Table S1**: List of primers used for RT-PCR and qPCR amplification of Ankrd2 and GAPDH fragments;

**Table S2**: Average Ct ± SD values for *ANKRD2* and *GAPDH* genes in cell lines derived from human osteoblasts (hFOB) and human OS.
