# Supplementary material for: Ectopic Expression of Ankrd2 Affects Proliferation, Motility and Clonogenic Potential of Human Osteosarcoma Cells
Source: Cancers (Basel). 2021 Jan 6;13(2):174. doi: 10.3390/cancers13020174 (PMC7825408; doi:10.3390/cancers13020174)
Supplement: Supplementary file 1 [file cancers-13-00174-s001.zip › Supplementary files/Supplementary Materials in separate file.docx]

**Supplementary Materials:** The following are available online at [www.mdpi.com/xxx/s1](http://www.mdpi.com/xxx/s1),

**Figure S1: Validation of the efficacy of the anti Ankrd2-phospho-Ser99 antibody;**

**Figure S2: Immunofluorescence analysis of endogenous Ankrd2 in U2OS and HOS cell lines;**

**Figure S3: Analysis of subcellular distribution of ectopically expressed Ankrd2~~wt~~ in Ankrd2-overexpressing clones from hFOB, U2OS, MG63, HOS and Saos2 cell lines.**

**Figure S4: Analysis of Ankrd2 expression level in clones of U2OS and HOS cells stably expressing a si-Ankrd2 transcript;**

**Figure S5: Effects of ectopic expression of Ankrd2 in a cell line derived from human rhabdomyosarcoma;**

**Figure S1B, S2A, S2B: original blots of Figure 1B, 2A and, 2B;**

**Table S1**: **List of primers used for RT-PCR and qPCR amplification of Ankrd2 and GAPDH fragments;**

**Table S2: Average Ct±SD values for *ANKRD2* and *GAPDH* genes in cell lines derived from human osteoblasts (hFOB) and human OS.**

**Figure S1**

**
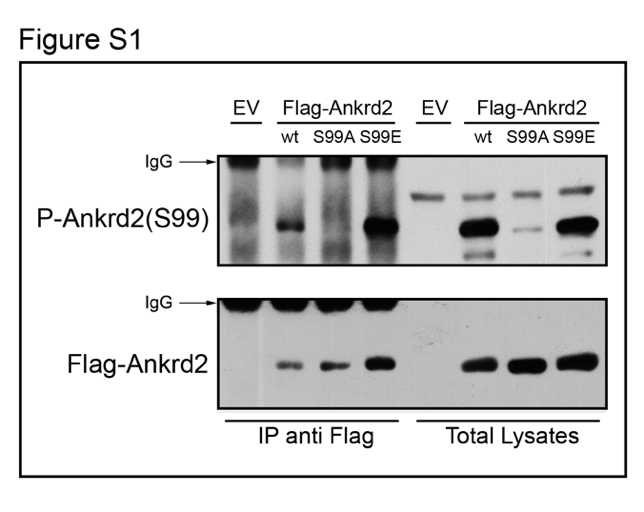
**

**Figure S1: Validation of the efficacy of the anti Ankrd2-phospho-Ser99 antibody.** HEK-293T cells were transfected with empty vector (EV) or with Flag-Ankrd2wt, Flag-Ankrd2(S99A), or Flag-Ankrd2(S99E). After 48 hours, cells were lysed and 800 μg of total lysates were subjected to immunoprecipitation with anti Flag (1μg, Sigma) and 30 μl of protein A/G agarose slurry (Santa Cruz Biotechnology) for 3 hours at 4°C. Co-immunoprecipitates and 20 μg of total lysates were resolved on a SDS-PAGE and analyzed with anti Ankrd2-phospho-Ser99 (P-Ankrd2, 1:2000 overnight at 4°C) and with anti Flag (1:1000 overnight at 4°C).

**Figure S2**


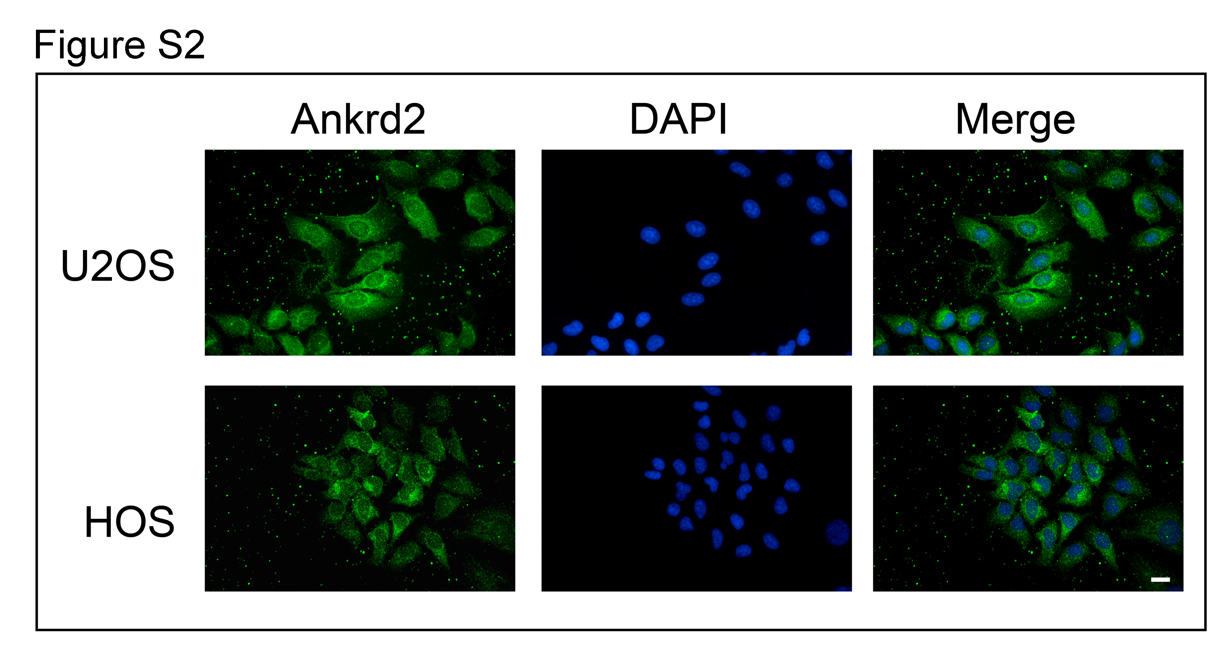


**Figure S2: Immunofluorescence analysis of endogenous Ankrd2 in U2OS and HOS cell lines.** U2OS and HOS cell lines, seeded on coverslips, were subjected to immunofluorescence analysis and stained with an anti-Ankrd2 primary antibody and FITC-conjugated secondary antibody to evaluate the localization of endogenous Ankrd2. A cytoplasmic as well as intranuclear staining was observed. Nuclei were counterstained with DAPI. Bar is 20μm.

**Figure S3**

**
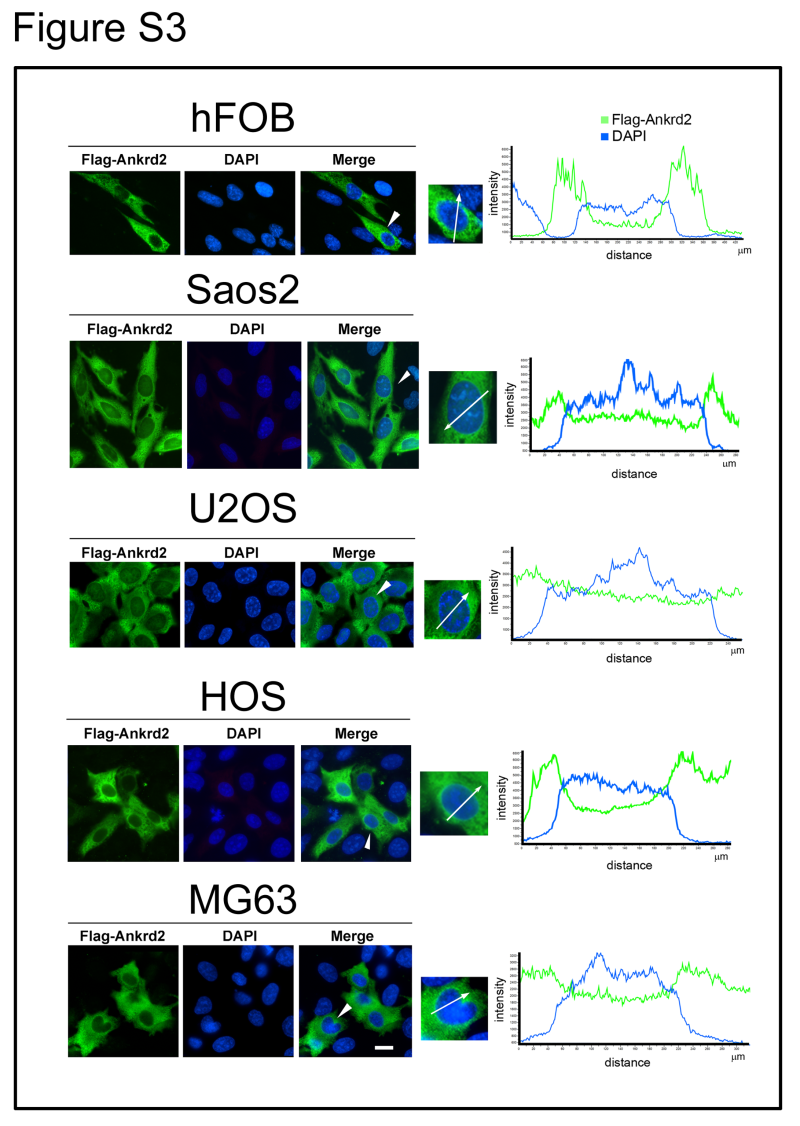
**

**Figure S3: Analysis of subcellular distribution of ectopically expressed Flag-Ankrd2wt in Ankrd2-overexpressing clones from hFOB, U2OS, MG63, HOS and Saos2 cell lines.** hFOB, Saos2, U2OS, HOS and MG63 cell lines expressing Flag-Ankrd2wt were seeded on coverslips and subjected to immunofluorescence analysis with an anti-Ankrd2 primary antibody and FITC-conjugated secondary antibody to evaluate the localization of Ankrd2. A cytoplasmic as well as intranuclear staining was observed. Nuclei were counterstained with DAPI. Bar is 20μm. Enlarged images show nuclei indicated by arrowheads. Graphs show the fluorescence intensity profile of Flag-Ankrd2 and DAPI signals along bars.

**Figure S4**

**
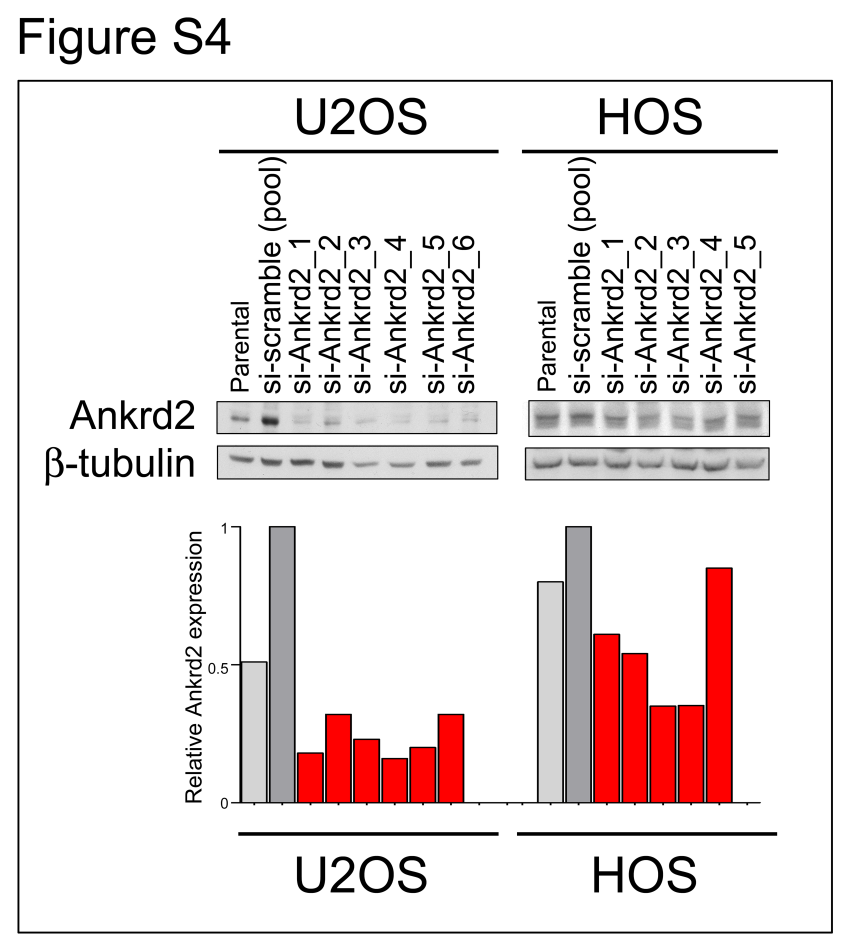
**

**Figure S4: Analysis of Ankrd2 expression level in clones of U2OS and HOS cells stably expressing a si-Ankrd2 transcript.** After more than one month of selection with the proper antibiotic (see Methods for details), six clones from U2OS and five from HOS cell lines were independently cultured and assayed for Ankrd2 expression level. For every cell line, total lysates from the corresponding parental cells transfected or not with a si-scramble were loaded together with the total lysates of si-Ankrd2 clones. β-tubulin was used as equal loading control.

**Figure S5**

**
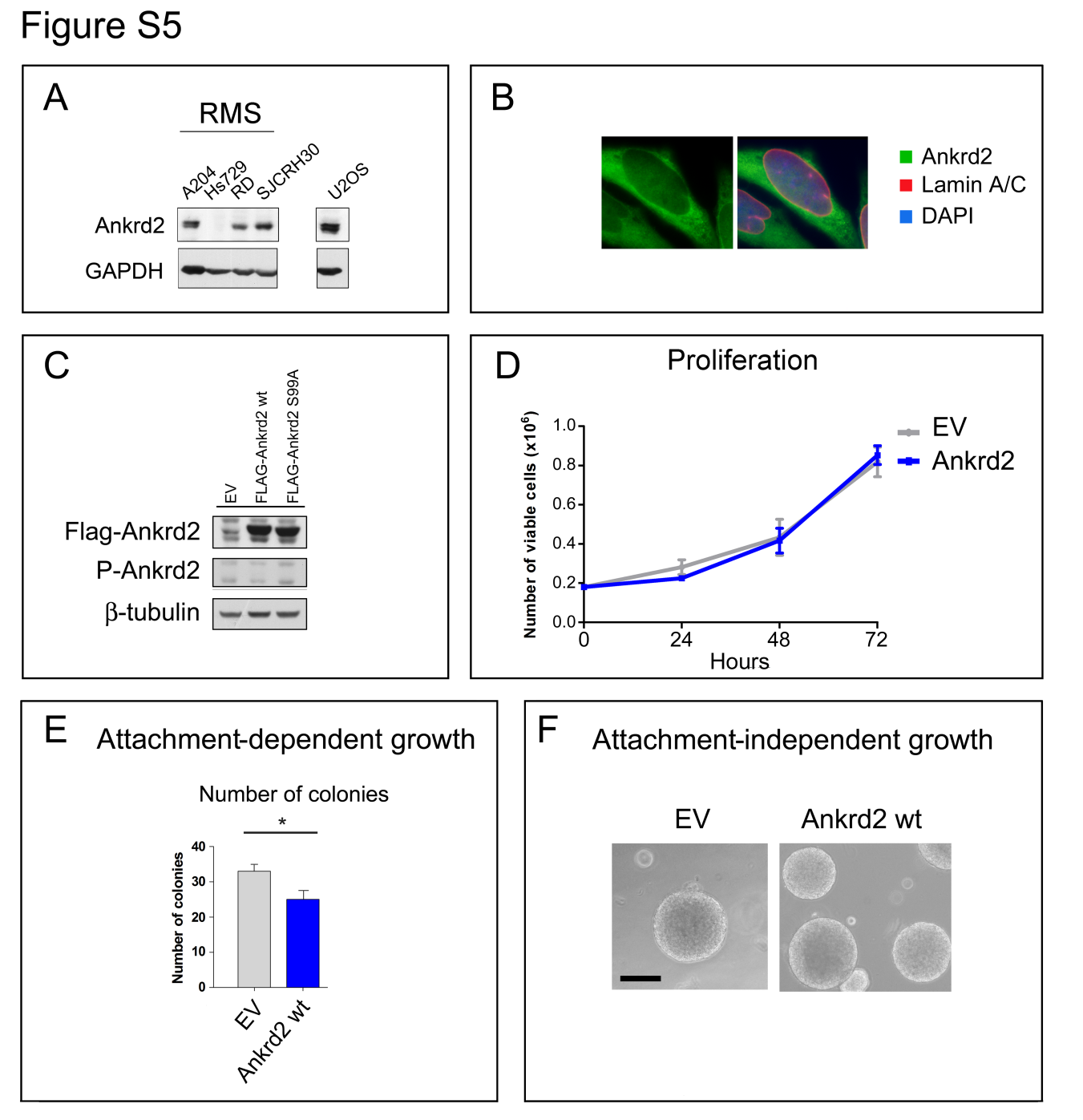
**

**Figure S5: Effects of ectopic expression of Ankrd2 in a cell line derived from human rhabdomyosarcoma. A)** A204, HS729, RD and SJCRH30 cell lines derived from human rhabdomyosarcoma (RMS) were lysed and assayed for basal expression of endogenous Ankrd2. A sample of U2OS total lysate was loaded as positive control. GAPDH was used as equal loading marker. **B)** Immunofluorescence analysis relative to the subcellular localization of overexpressed Ankrd2 in RD cells. Cells were seeded on coverslips, fixed, permeabilized and stained for Ankrd2 and lamin A/C. Bar is 20μm. Staining of lamin A/C revealed nuclear morphology defects not related to Ankrd2 expression. **C)** Analysis of the level of the expression and phosphorylation at Serine 99 of Ankrd2 forms in RD cells stably expressing Flag-Ankrd2wt or Flag-Ankrd2S99A or transfected with empty vector (EV). β-tubulin was used as equal loading control. **D)** Effect of ectopic expression of Flag-Ankrd2wt on proliferation of RD cells. RD cells overexpressing Flag-Ankrd2wt were seeded at a density of 2x10^5^ cells/well in six-well tissue culture plates. After 24, 48 and 72 hours from seeding, cells were individually collected and counted under light microscope. As control, the proliferation rate of RD cells transfected with empty vector (EV) was also evaluated. Data are representative of a minimum of three independent experiments. Data were analyzed and graphed using the GraphPad Prism software. **E)** RD cells overexpressing Flag-Ankrd2wt or transfected with empty vector (EV) were seeded at a density of 13.3/cm^2^ of culture dish and allowed to grow for two weeks. Colonies with >50 cells were counted and graphed using the GraphPad Prism software. The clonogenic potential of parental RD cells was reduced by ectopic expression of Flag-Ankrd2wt. Statistical analysis was performed using an unpaired two-tail Student’s t test. **p* < 0.01. **F**) RD cells overexpressing Flag-Ankrd2wt, or transfected with empty vector (EV) were seeded at a density of 2×10^5^ cells/35 cm^2^ of culture dish in complete media containing methyl-cellulose and allowed to grow. Spheroids were observed under light microscope. No statistically significant differences in the forming rate or dimension of spheroids were observed. Representative images were shown. Bar is 100μm

**Table S1**

|  | Forward Primer | Reverse Primer | amplicon size (bp) |
| --- | --- | --- | --- |
| *ANKRD2* | CGGTTATGGACGGCACCAT | CTTCTCATCCTCCAGCACCA | 155 |
| *GAPDH* | GTGAAGGTCGGAGTCAACG | TGAGGTCAATGAAGGGGTC | 112 |

**Table S1**: List of primers used for RT-PCR and qPCR amplification of Ankrd2 and GAPDH fragments.

**Table S2**

|  | ***ANKRD2*** | | ***GAPDH*** | |
| --- | --- | --- | --- | --- |
|  | avg Ct | SD | avg Ct | SD |
| hFOB | 29.919 | 0.851 | 14.436 | 0.216 |
| Saos2 | 31.118 | 0.341 | 14.658 | 0.062 |
| U2OS | 26.886 | 0.542 | 14.483 | 0.151 |
| HOS | 29.269 | 0.359 | 14.644 | 0.179 |
| MG63 | 30.209 | 0.733 | 14.320 | 0.109 |

**Table S2**: Average Ct±SD values for *ANKRD2* and *GAPDH* genes in cell lines derived from human osteoblasts (hFOB) and human OS (Saos2, U2OS, HOS and MG63).
