# Supplementary material for: Ectopic Expression of Ankrd2 Affects Proliferation, Motility and Clonogenic Potential of Human Osteosarcoma Cells
Source: Cancers (Basel). 2021 Jan 6;13(2):174. doi: 10.3390/cancers13020174 (PMC7825408; doi:10.3390/cancers13020174)
Supplement: Supplementary file 1 [file cancers-13-00174-s001.zip › Supplementary files/Rev_Piazzi et al_Table S1.docx]

|  | forward primer | reverse primer | amplicon size (bp) |
| --- | --- | --- | --- |
| *ANKRD2* | CGGTTATGGACGGCACCAT | CTTCTCATCCTCCAGCACCA | 155 |
| *GAPDH* | GTGAAGGTCGGAGTCAACG | TGAGGTCAATGAAGGGGTC | 112 |
