# Supplementary figures and images for: Ectopic Expression of Ankrd2 Affects Proliferation, Motility and Clonogenic Potential of Human Osteosarcoma Cells
Source: Cancers (Basel). 2021 Jan 6;13(2):174. doi: 10.3390/cancers13020174 (PMC7825408; doi:10.3390/cancers13020174)

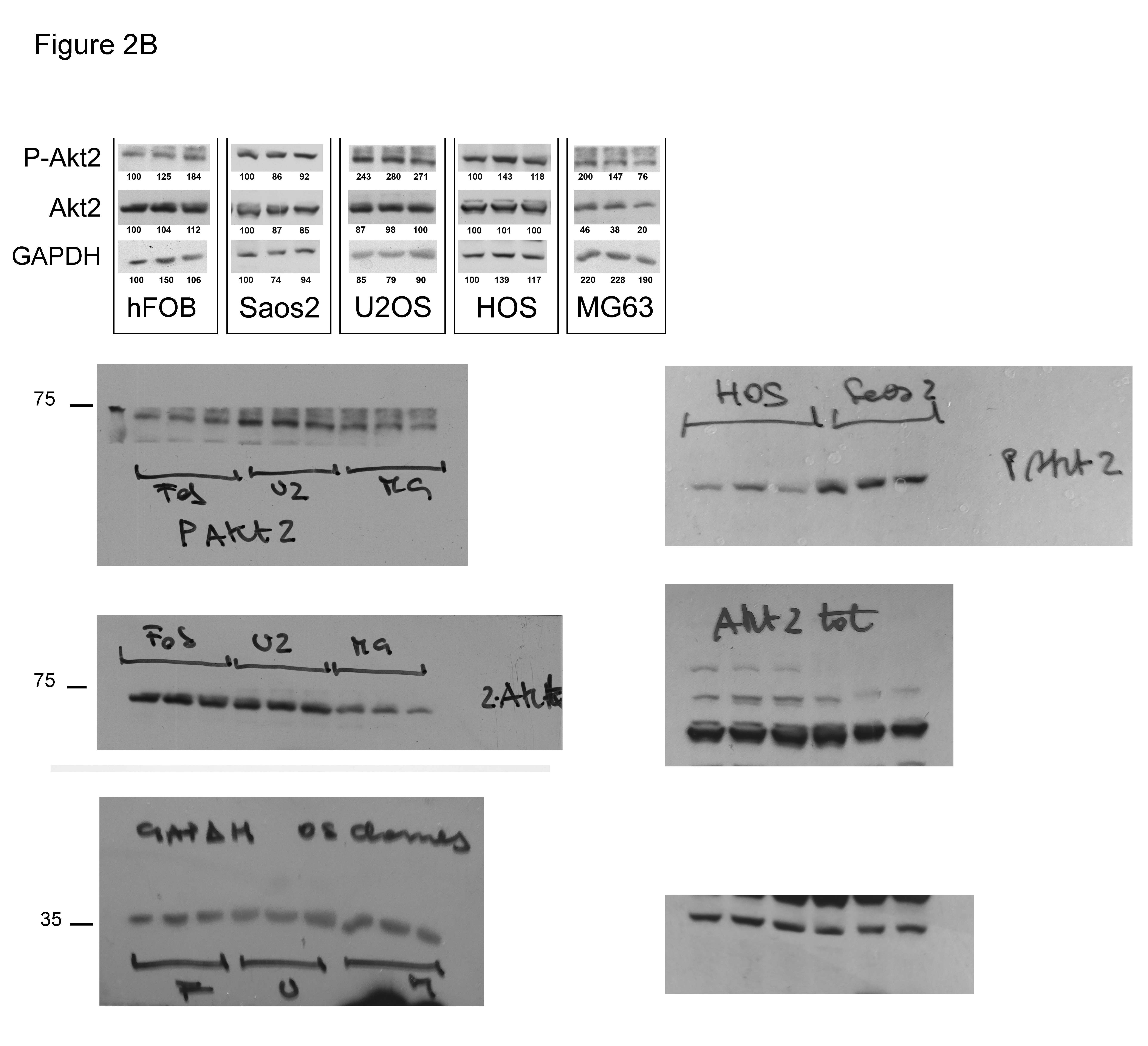

Supplement: Supplementary file 1 [file cancers-13-00174-s001.zip › Supplementary files/Piazzi et al_Figure S2B.tif]

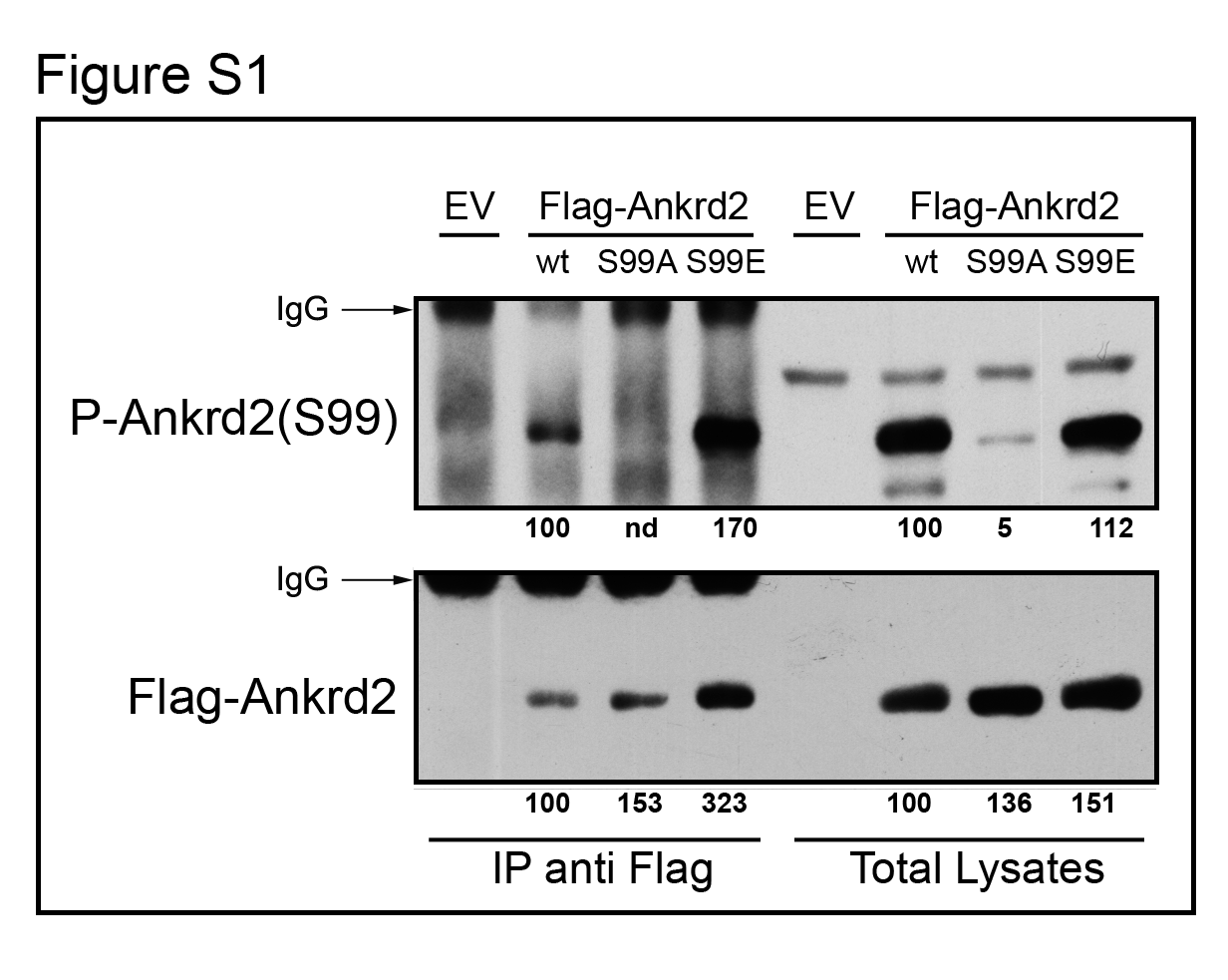

Supplement: Supplementary file 1 [file cancers-13-00174-s001.zip › Supplementary files/Rev_Piazzi et al_Figure S1.tif]

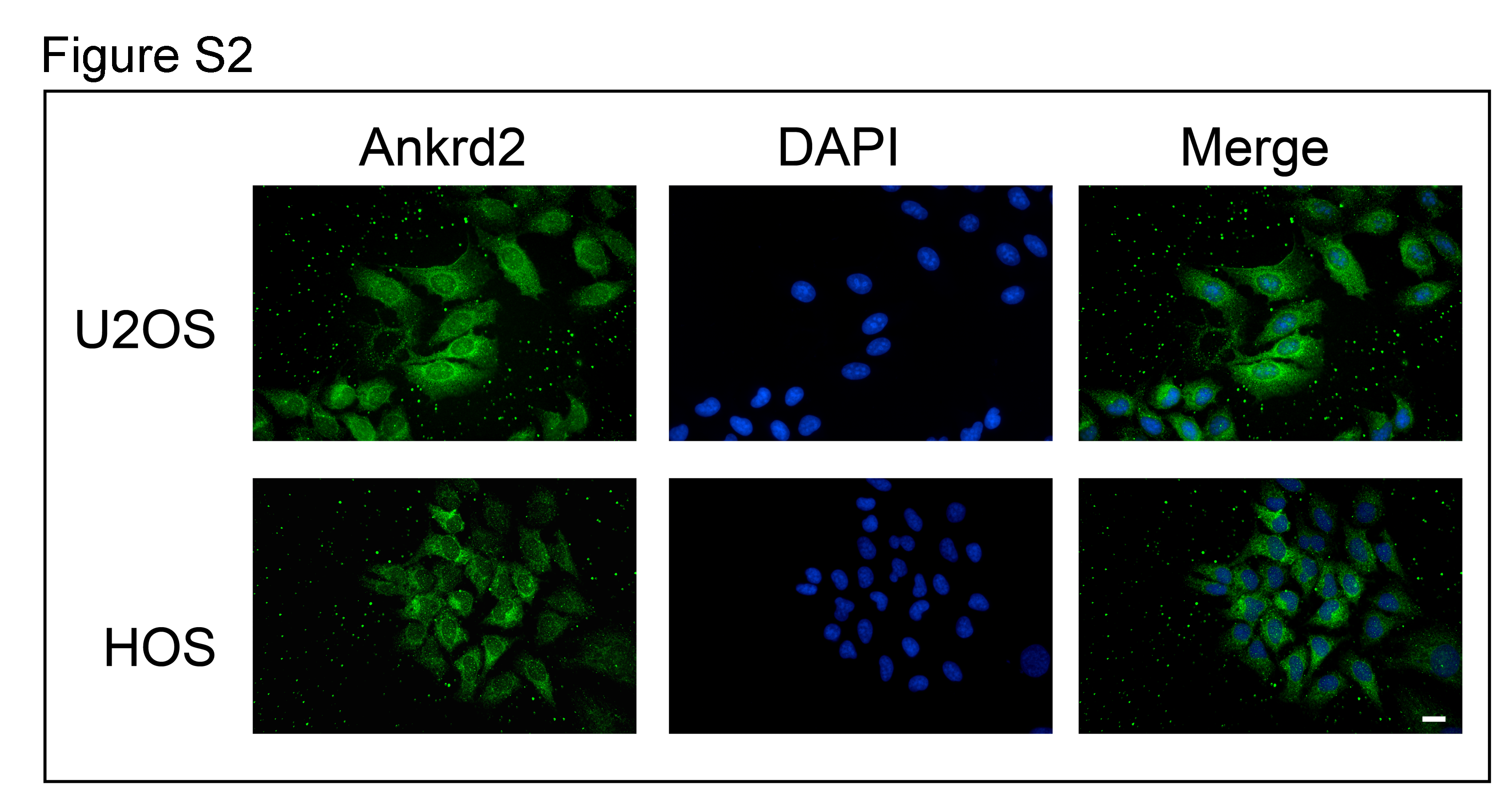

Supplement: Supplementary file 1 [file cancers-13-00174-s001.zip › Supplementary files/Rev_Piazzi et al_Figure S2.tif]

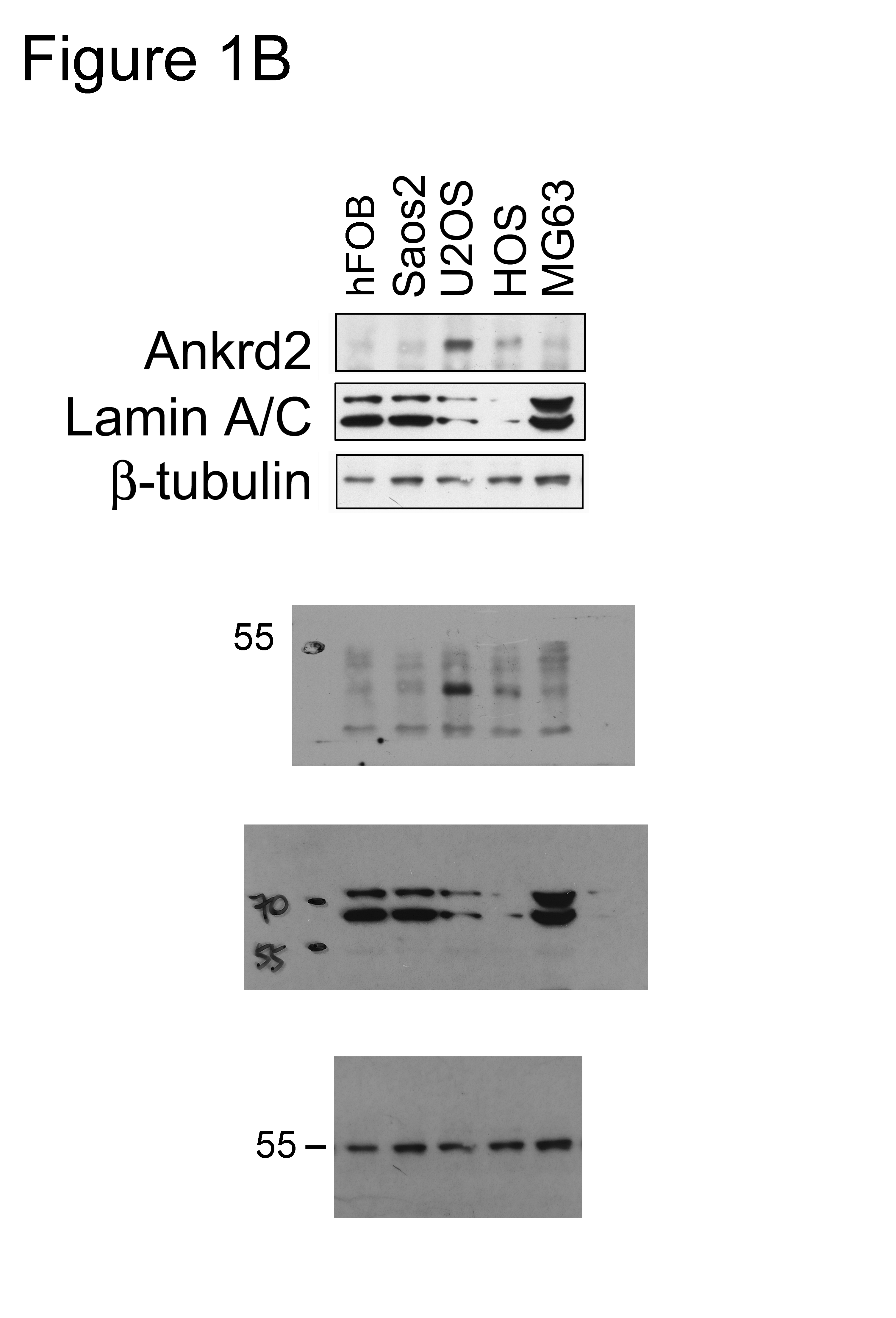

Supplement: Supplementary file 1 [file cancers-13-00174-s001.zip › Supplementary files/Piazzi et al_Figure S1B.tif]

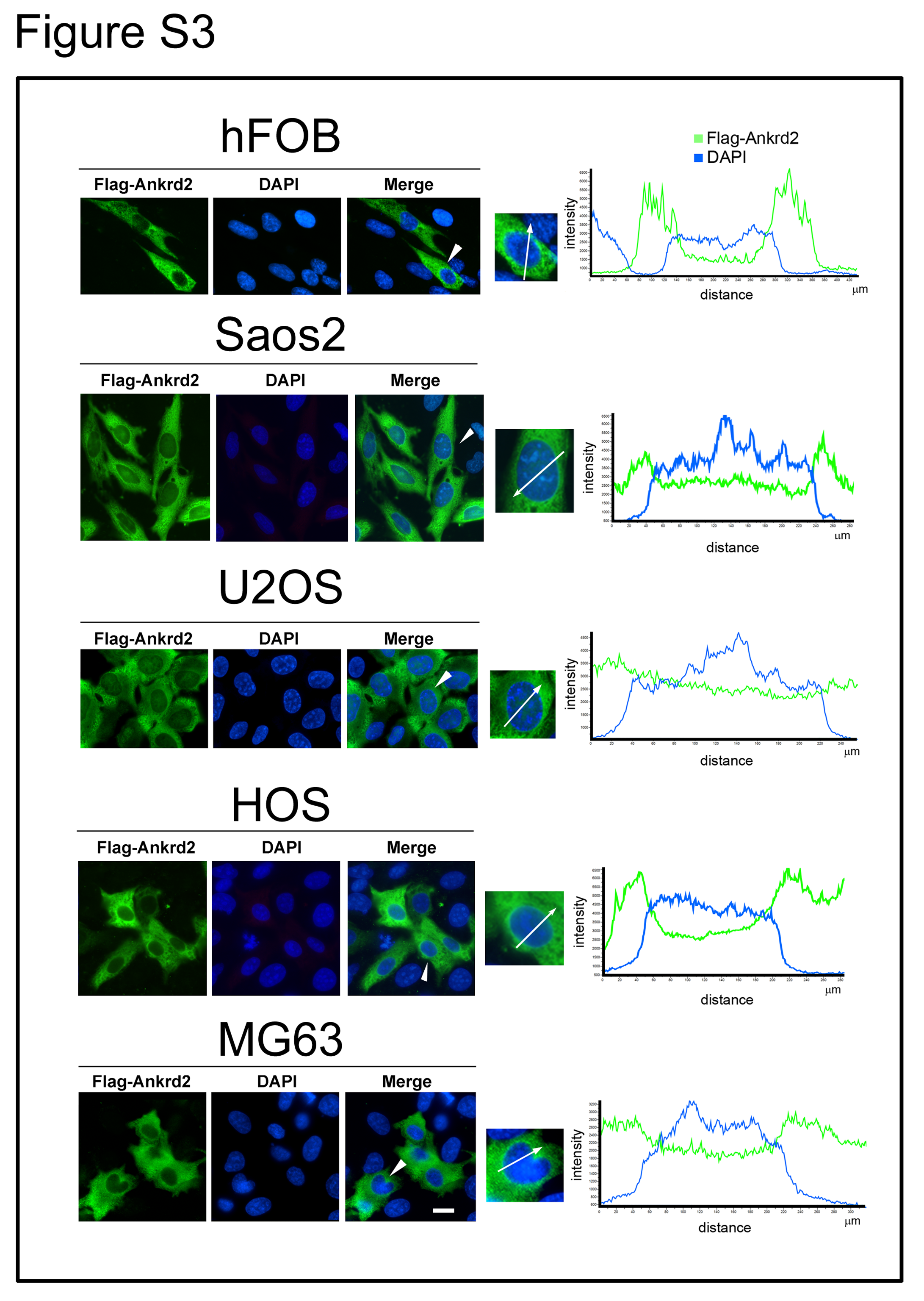

Supplement: Supplementary file 1 [file cancers-13-00174-s001.zip › Supplementary files/Rev_Piazzi et al_Figure S3.tif]
